# Gender Differences in Job Satisfaction and Work-Life Balance Among Chinese Physicians in Tertiary Public Hospitals

**DOI:** 10.3389/fpubh.2021.635260

**Published:** 2021-05-10

**Authors:** Dan Liu, Yinuo Wu, Feng Jiang, Mingxiao Wang, Yuanli Liu, Yi-Lang Tang

**Affiliations:** ^1^School of Health Policy and Management, Chinese Academy of Medical Sciences & Peking Union Medical College, Beijing, China; ^2^Chinese Academy of Medical Sciences & Peking Union Medical College, Beijing, China; ^3^Institute of Healthy Yangtze River Delta, Shanghai Jiao Tong University, Shanghai, China; ^4^Department of Cardiology, Emergency General Hospital, Beijing, China; ^5^Department of Psychiatry and Behavioral Sciences, Emory University, Atlanta, GA, United States; ^6^Atlanta VA Medical Center, Decatur, Georgia, GA, United States

**Keywords:** job satisfaction, work-life balance, gender difference, hospitals, China

## Abstract

**Background:** Gender has been associated with job-related experience, including job satisfaction and work-life balance. This study aimed to identify gender differences in job satisfaction and work-life balance among Chinese physicians in a large, nationally representative sample.

**Methods:** A national cross-sectional survey was conducted between March 18 and 31, 2019, using an anonymous online questionnaire. The questionnaire included the short-form MSQ (Chinese version) and a work-life balance item. The demographic and job-related factors were also collected.

**Findings:** In total, 22,128 physicians (9,378 males and 12,750 females) from 144 tertiary public hospitals completed the survey. The overall MSQ score (job satisfaction) was 70.31 ± 12.67, and it was 69.89 ± 13.24 in males, and 70.63 ± 12.22 in females, respectively (*p* < 0.001). Only 931 (4.21%) physicians were very satisfied with WLB (421 males, 510 females), and 2,534 (11.45%) were rated as satisfied. Age, education, monthly income, working hours, specialty, and professional titles were significantly associated with job satisfaction; while number of children, specialty, professional titles, monthly income, age, working hours were significantly associated with WLB. No significant gender differences were observed in job satisfaction or WLB after controlling confounding factors (both *p* > 0.05).

**Interpretation:** While many demographic and work-related factors are significantly associated with job satisfaction and WLB, we found no significant gender differences, which is different from many other studies. To improve Chinese physicians' job satisfaction and work-life balance, interventions should be focused on certain specialties and on other modifiable factors, such as income, working hours.

## Introduction

Job satisfaction can be defined as the attitudes resulting from one's job experience (1, 2). Previous studies have identified a series of factors associated with physicians' job satisfaction, such as age, gender, marital status, professional title, and educational background etc (3–9). Many studies have examined gender differences of job satisfaction in physicians. While some did not find significant gender differences (10–15), and a few other studies even found the opposite, that male physician reported higher job satisfaction (16, 17).

Work-life balance (WLB) is a common concept with no universally accepted definition. It can be roughly defined as the state of equilibrium degree of how a person equally prioritizes the demands of his/her career and the needs of his/her personal life, with a minimum role conflict at work and at home life (18). A good WLB improves job satisfaction, psychosocial well-being, and overall quality of life (19, 20). On the contrary, a work-life imbalance (bad WLB) often leads to psychological strain, lower life and job satisfaction, depression, burnout, and family conflict, and other health problems (21–25).

Gender is associated with many job-related factors, and the relationship between gender and WLB has recently attracted much interest. As gender refers to the culturally defined roles and responsibilities in given settings, traditional gender roles prescribe different emphases for men and women: work for men and family responsibility for women (26). Although gender systems could be equitable, gender inequality in WLB have often been reported in past decades. Many studies showed that gender differences existed in job satisfaction with medical practice. In most cultures, especially Asian cultures, female workers are expected to share more household responsibilities than their male counterparts. They are more likely to reach the “glass ceiling” of career (27, 28). A series of studies demonstrated that gender plays a vital role in WLB (29–33).

In the healthcare field, several previous studies investigated gender difference in WLB, but got inconsistent results (34–39). One study showed that WLB varied significantly among healthcare specialties, length of work time and work settings (18). The situation also understandably varies across different cultures, and some believe this situation may be worse in Eastern countries due to the long history of sexism (40). However, there have been few studies examining gender differences in WLB among Chinese physicians. Therefore, this study aimed to investigate gender differences in physicians' job satisfaction and WLB in Chinese tertiary public hospitals.

## Data and Methods

### Study Design and Samples

The study was a part of a national survey conducted in 2019, the China National Healthcare Improvement Initiative Survey (41). The National Health Commission of China approved and sponsored this survey. The project was completed between March 18 to 31, 2019. We purposely involved 144 tertiary public hospitals in the capital cities of each province in mainland China, including 59 general hospitals, 37 Traditional Chinese Medicine (TCM) hospitals, 33 maternal and children's hospitals, five stomatological hospitals, four cancer hospitals, and six other specialty hospitals. These hospitals accounted for 6.45% of all the tertiary hospitals, delivered 12.28% of the inpatient care among tertiary hospitals, 10.90% of all physicians in tertiary hospitals (42). In the Chinese healthcare system, tertiary hospitals play a critical role.

Based on their employee's ID codes on the hospital staff lists, physicians were sampled through a systematic sampling method in each participating hospital. We invited 170 physicians from each hospital to participate. The survey was conducted anonymously through WeChat, a widely used online social media application in China.

### Ethics Statement

The Ethics Committee (IEC) of the Emergency General Hospital in Beijing approved the study protocol. All participated physicians signed the informed consent before they proceeded to respond to the questionnaires. The informed consent statement explained the purpose of the survey, ensured that the data would be de-identified before analysis and that the hospitals' administrators would have no access to their responses.

### Measures

The online questionnaire collected their demographic information, including gender, age, marital status, number of children, educational level, department (specialty), professional title, average monthly income, average working hours per week, hospital type, and location (geographical region).

Job satisfaction was measured through the short version of the Minnesota Satisfaction Questionnaire (MSQ) (Chinese version), which has been widely used and has demonstrated good reliability and validity (43, 44). Work-life balance was measured using the following question: “How are you satisfied with the balance of your work and your family?” (30, 36). They were used as the outcome measure in this study.

The items for MSQ and WLB were 5-point Likert scale responses: very dissatisfied-1, dissatisfied-2, neither-3, satisfied-4, and very satisfied-5. The responses for WLB were dichotomized into two groups: Dissatisfied WLB (very dissatisfied/dissatisfied) and non-Dissatisfied WLB (neither/satisfied/very satisfied).

### Statistical Analysis

Descriptive analyses were conducted for the variables. Continuous variables were shown with mean and standard deviation, while categorical variables were shown with numbers and percentages. Ages, average working hours per week, MSQ scores were treated as continuous variables. Ages, average working hours between male and female physicians were tested by ANOVA. Chi-square tests were used to examine other characteristics.

As the physicians were nested in 144 hospitals, the null model demonstrated that the intra-class correlation was more than 10%, so we used multi-level linear and logistic regression models to examine gender differences in MSQ and WLB, respectively (45). Participants with missing data were rare (<1%) and were excluded from the analyses.

All statistical analyses were conducted using the statistical software Stata 15 (StataCorpLP, College Station, TX, USA). All statistical analysis tests were two-sided, and the statistical significance was defined as *p* < 0.05.

## Results

### Description of Sample Characteristics and Related Factors

In total, 24,480 physicians were invited to participate, and 22,416 responded (response rate = 91.57%). After removing 288 participants with incomplete data, data from 22,128 physicians (22128/22416, 98.72%) were included in the final analysis. Their socio-demographic and job-related characteristics are shown in Table 1. There were 9,378 male (42.4%) and 12,750 (57.6%) female physicians. The average weekly working hours were 55.85 ± 15.10 h in this sample. The gender differences in demographic factors (age, marriage status, number of children, education), work-related factors (specialty, professional title, income, hospital type, working-hours, and regions) were all significant (Table 1).

**Table 1:** Characteristics of physicians in tertiary public hospitals.

**Characteristic**	**Total (*****N*** **=** **22128)**		**Male (*****N*** **=** **9378)**		**Female (*****N*** **=** **12750)**		**Statistics**	
	***N***	**%**	***N***	**%**	***N***	**%**	*****χ***^2^**	***p***
Marital status							111.164	** <0.001**
Single	3244	14.66	1135	12.10	2109	16.54		
Married	18261	82.52	8033	85.66	10228	80.22		
Divorced or widowed	623	2.82	210	2.24	413	3.24		
Children							185.063	** <0.001**
None	5822	26.31	2111	22.51	3711	29.11		
One	12782	57.76	5489	58.53	7293	57.20		
More than one	3524	15.93	1778	18.96	1746	13.69		
Educational level^*^							206.736	** <0.001**
Bachelor degree or below	5628	25.43	2256	24.06	3372	26.45		
Master's degree	10440	47.18	4084	43.55	6356	49.85		
Doctorate degree	6060	27.39	3038	32.39	3022	23.70		
Department/Specialty							2.6e+03	** <0.001**
Internal medicine	6576	29.72	2369	25.26	4207	33.00		
Surgery	7281	32.90	4582	48.86	2699	21.17		
Ob/Gyn^**^	3099	14.00	368	3.92	2731	21.42		
Pediatrics	2229	10.07	760	8.10	1469	11.52		
Emergency	1030	4.65	431	4.60	599	4.70		
Miscellaneous others^***^	1913	8.65	868	9.26	1045	8.20		
Professional title							188.044	** <0.001**
Junior	6247	28.23	2266	24.16	3981	31.22		
Middle	8202	37.07	3445	36.73	4757	37.31		
Associate senior	4889	22.09	2359	25.15	2530	19.84		
Senior	2790	12.61	1308	14.82	1482	11.62		
Average monthly income							173.051	** <0.001**
<5000 RMB	5917	26.74	2267	24.17	3650	28.63		
5000–9999 RMB	6252	28.25	2468	26.32	3784	29.68		
10,000–20,000 RMB	7290	32.94	3264	34.80	4026	31.58		
>20,000 RMB	2669	12.06	1379	14.70	1290	10.12		
Hospital type							638.724	** <0.001**
General hospitals	8865	40.06	4373	46.63	4492	35.23		
TCM general hospitals	6098	27.56	2829	30.17	3269	25.64		
Specialty hospitals	7165	32.38	2176	23.20	4989	39.13		
Location/Region							17.443	** <0.001**
East China	9553	43.17	4128	44.02	5425	42.55		
Central China	5240	23.68	2285	24.37	2955	23.18		
West China	7335	33.15	2965	31.62	4370	34.27		
	**Mean**	**SD**	**Mean**	**SD**	**Mean**	**SD**	***T***	***p***
Age (years)	37.94	8.13	38.86	8.15	37.27	8.05	14.516	** <0.001**
Working hours/week	55.85	15.10	57.31	15.68	54.77	14.56	12.466	** <0.001**

* *In China, medical school graduates are awarded with a bachelor degree of medicine (similar to the European and Russian systems). Some obtained a master's or doctorate degree in addition to their medical degree.*

** *Ob/Gyn: obstetrics-gynecology.*

*** *Including oncology department, rehabilitation department, reproductive health department, geriatrics department, etc.*

*Bold value for p < 0.05*.

The MSQ score was 70.31 ± 12.67 overall, and 69.89 ± 13.24 in male physicians, 70.63 ± 12.22 in female physicians, respectively (*p* < 0.001). Univariate analysis demonstrated that all other demographic factors (age, marriage status, number of children, education) and work-related factors (specialty, professional title, income, hospital type, working-hours, and regions) were significantly associated with MSQ scores (Table 2).

**Table 2:** Univariate analysis of MSQ.

	**Total (*****N*** **=** **22128)**		**Statistics**	
**Characteristic**	**Mean**	**SD**	***F/T***	***p***
Gender			18.25	** <0.001**
Male	69.89	13.24		
Female	70.63	12.22		
Marital status			4.43	**0.012**
Single	70.54	12.55		
Married	70.32	12.67		
Divorced or widowed	68.89	13.19		
Children			4.83	**0.008**
None	70.67	12.44		
One	70.09	12.66		
More than one	70.53	13.06		
Educational level			61.33	** <0.001**
Bachelor degree or below	69.18	12.56		
Master's degree	70.12	12.62		
Doctorate degree	71.71	12.73		
Department/specialty			13.61	** <0.001**
Internal medicine	69.50	12.44		
Surgery	70.26	12.79		
Ob/Gyn	71.62	13.13		
Pediatrics	70.32	12.27		
Emergency	70.45	12.73		
Miscellaneous	71.12	12.47		
Professional title			55.49	** <0.001**
Junior	71.23	12.79		
Middle	69.61	12.59		
Associate senior	69.19	12.52		
Senior	72.31	12.55		
Average monthly income			152.87	** <0.001**
<5000 RMB	68.91	12.44		
5000–9999 RMB	68.90	12.47		
10,000–20,000 RMB	71.22	12.54		
>20,000 RMB	74.28	12.91		
Hospital type			121.05	** <0.001**
General hospitals	69.39	12.34		
TCM general hospitals	69.41	12.31		
Specialty Hospitals	72.22	13.15		
Location/region			64.02	** <0.001**
East China	71.05	12.48		
Central China	70.88	13.12		
West China	68.95	12.48		
	**Correlation coefficient**		***p***	
Age (years)	−0.0208		**0.002**	
Working hours/week	−0.2057		** <0.001**	

*Bold value for p < 0.05*.

In the multi-level linear regression analysis model, we found that physicians with doctorate degrees, in Ob/Gyn and “Miscellaneous departments” (which included oncology department, rehabilitation department, reproductive department, geriatrics department, etc.), with senior professional titles, with a monthly income of more than 10,000 RMBs, in specialty hospitals, had higher MSQ scores, with middle and associate senior professional titles, elder, longer working hours were significantly associated with lower MSQ scores. Although there was a trend that female physicians had a little higher MSQ scores than males, it was not statistically significant (*p* = 0.057) (Table 3).

**Table 3:** Multi-level linear regression examining factors associated with MSQ.

	**Coef**.	**95.0% CI (Lower)**	**95.0% CI (Upper)**	***p***
Gender (ref. Male)	0.32	−0.01	0.65	0.057
Marital status (ref. Single)				
Married	0.42	−0.17	1.01	0.162
Divorced or widowed	−0.74	−1.79	0.32	0.170
Children(ref. None)				
One	−0.34	−0.85	0.17	0.193
More than one	−0.14	−0.75	0.47	0.650
Educational level (ref. Bachelor's degree or below)				
Master's degree	0.31	−0.11	0.73	0.143
Doctorate degree	1.18	0.65	1.72	** <0.001**
Department(ref. internal medicine)				
Surgery	0.26	−0.16	0.68	0.225
Ob/Gyn	0.60	0.01	1.18	**0.046**
Pediatrics	0.27	−0.31	0.86	0.353
Emergency	0.39	−0.37	1.15	0.314
Miscellaneous	1.17	0.58	1.76	** <0.001**
Professional title (ref. Junior)				
Middle	−1.60	−2.05	−1.15	** <0.001**
Associate senior	−1.43	−2.08	−0.77	** <0.001**
Senior	1.88	0.98	2.78	** <0.001**
Average monthly income (ref. <5000 RMB)				
5000–9999 RMB	−0.13	−0.54	0.29	0.547
10,000–20,000 RMB	1.41	0.99	1.84	** <0.001**
>20,000 RMB	3.17	2.55	3.79	** <0.001**
Hospital type (ref. General hospitals)				
TCM general hospitals	0.01	−1.85	1.86	0.993
Specialty Hospitals	1.82	0.08	3.56	**0.040**
Location(ref. East China)				
Central China	1.00	−0.87	2.87	0.293
West China	−0.17	−1.90	1.56	0.847
Age	−0.14	−0.18	−0.11	** <0.001**
Working hours/week	−0.15	−0.16	−0.14	** <0.001**

*Bold value for p < 0.05*.

In the whole sample, only 931 (4.21%) physicians were very satisfied with WLB (421 males, 510 females), 2,534 (11.45%) rated as satisfied (1,030 males and 1,504 females), 8,140 (36.79%) rated as neither satisfied nor dissatisfied (3,295 males and 4,845 females), 6,288 (28.42%) as dissatisfied (2,624 males and 3,664 females), 4,235 (19.14%) as very dissatisfied (2,008 males and 2,227 females). In short words, only 15.70% of physicians were satisfied or very satisfied with their WLB.

After regrouping the reported WLB into a categorical variable, 10,523 (47.56%) were classified as dissatisfied with WLB and 11,605 (52.44%) as non-dissatisfied with WLB. Among them, 49.39% of male and 46.20% of female physicians were classified as dissatisfied WLB, respectively (*p* < 0.001). All other demographic and work-related factors were significantly associated with WLB in univariate analysis (details see Table 4).

**Table 4:** Univariate analysis of WLB.

	**Not bad WLB (11605)**		**Bad WLB (10523)**		**Statistics**	
**Characteristic**	***N***	**%**	***N***	**%**	*****χ***^2^**	***p***
Gender					22.02	** <0.001**
Male	4746	50.61	4632	49.39		
Female	6859	53.80	5891	46.20		
Marital status					22.53	** <0.001**
Single	1826	56.29	1418	43.71		
Married	9455	51.78	8806	48.22		
Divorced or widowed	324	52.01	299	47.99		
Children					100.16	** <0.001**
None	3322	57.06	2500	42.94		
One	6643	51.97	6139	48.03		
More than one	1640	46.54	1884	53.46		
Educational level					38.27	** <0.001**
Bachelor degree or below	3147	55.92	2481	44.08		
Master's degree	5393	51.66	5047	48.34		
Doctorate degree	3065	50.58	2995	49.42		
Department					71.43	** <0.001**
Internal Medicine	3284	49.94	3292	50.06		
Surgery	3940	54.11	3341	45.89		
Ob/Gyn	1582	51.05	1517	48.95		
Pediatrics	1096	49.17	1133	50.83		
Emergency	593	57.57	437	42.43		
Miscellaneous	1110	58.02	803	41.98		
Professional title					139.98	** <0.001**
Junior	3506	56.12	2741	43.88		
Middle	3972	48.43	4230	51.57		
Associate senior	2482	50.77	2407	49.23		
Senior	1645	58.96	1145	41.04		
Average monthly income					17.83	** <0.001**
<5,000 RMB	3119	52.71	2798	47.29		
5,000–9,999 RMB	3188	50.99	3064	49.01		
10,000–20,000 RMB	3808	52.24	3482	47.76		
>20,000 RMB	1490	55.83	1179	44.17		
Hospital type					90.93	** <0.001**
General hospitals	4306	48.57	4559	51.43		
TCM general hospitals	3315	54.36	2783	45.64		
Specialty Hospitals	3984	55.60	3181	44.40		
Location					65.14	** <0.001**
East China	5251	54.97	4302	45.03		
Central China	2778	53.02	2462	46.98		
West China	3576	48.75	3759	51.25		
	**Mean**	**SD**	**Mean**	**SD**	***T***	***p***
Age(years)	38.25	8.70	37.61	7.44	5.85	** <0.001**
Working hours/week	50.98	12.91	61.21	15.51	53.50	** <0.001**

*Bold value for p < 0.05*.

In the multi-level logistic regression model, we found physicians with one (OR = 1.52) or more than one child (OR = 1.77), middle (OR = 1.36) or associate senior professional title (OR = 1.35), monthly income of 5,000–9,999 RMBs (OR = 1.10), longer working hours/week (OR = 1.06) were more likely to have dissatisfied WLB. While physicians in surgery (OR = 0.85) or “Miscellaneous departments” (OR = 0.80), older (OR = 0.99) were less likely to have dissatisfied WLB. Similar to the analysis with job satisfaction, we failed to observe significant gender differences in WLB after controlling for confounding factors (Table 5).

**Table 5:** Multi-level Logistic regression examining factors associated with WLB (Dissatisfied WLB vs. non-Dissatisfied WLB).

	**OR**	**95.0% CI (Lower)**	**95.0% CI (Upper)**	***p***
Gender (ref. Male)	0.98	0.92	1.04	0.520
Marriage status (ref. Single)				
Married	0.92	0.82	1.03	0.149
Divorced or widowed	1.05	0.86	1.29	0.627
Children(ref. None)				
One	1.52	1.38	1.68	** <0.001**
More than one	1.77	1.57	1.99	** <0.001**
Educational level (ref. Bachelor degree or below)				
Master's degree	1.05	0.96	1.14	0.279
Doctorate degree	1.10	0.99	1.21	0.079
Department (ref. Internal Medicine)				
Surgery	0.85	0.79	0.93	** <0.001**
Ob/Gyn	0.99	0.89	1.11	0.897
Pediatrics	1.11	0.99	1.24	0.082
Emergency	0.97	0.84	1.13	0.732
Miscellaneous	0.80	0.72	0.90	** <0.001**
Professional title (ref. Junior)				
Middle	1.36	1.24	1.48	** <0.001**
Associate senior	1.35	1.19	1.53	** <0.001**
Senior	1.10	0.93	1.31	0.265
Average monthly income(ref. <5000 RMB)				
5,000–9,999 RMB	1.10	1.01	1.19	**0.026**
10,000–20,000 RMB	1.08	0.99	1.17	0.073
>20,000 RMB	1.00	0.88	1.12	0.951
Hospital type(ref. General hospitals)				
TCM general hospitals	0.91	0.75	1.10	0.326
Specialty hospitals	0.90	0.75	1.08	0.268
Location(ref. East China)				
Central China	0.88	0.73	1.07	0.213
West China	1.10	0.91	1.31	0.327
Age (years)	0.99	0.98	1.00	**0.015**
Working hours/week	1.06	1.05	1.06	** <0.001**

*Bold value for p < 0.05.*

*Dissatisfied WLB was defined as 1 and non-Dissatisfied WLB as 0*.

## Discussion

To our best knowledge, this study was among the first studies focusing on gender differences in job satisfaction and WLB based on a nationally representative physician sample in China, covering a wide range in terms of specialty, hospital types, and geographical regions. While we replicated some of the findings reported by others, one unique finding is that no significant gender differences were found in physician job satisfaction and WLB.

Gender differences are a longstanding phenomenon and an important research topic, in which job satisfaction and WLB are two aspects that have gained research attention in recent years. Job satisfaction describes the level of contentment and fulfillment that employees derive from their jobs. Studies suggested that different job expectations, values and many other factors may contribute to the differences in job satisfaction between men and women (17). In the healthcare field, studies of WLB have mostly centered on “role” and the conflicts between work and family as the main sources of poor WLB. The society often has double expectations for women, this is particularly true in China, where most people believe in the traditional sex role assignment. On one side, women are expected to perform well in their career; on the other side, they are also expected to be a “good wife and good mother” and take most household responsibilities. Therefore, career women often suffer more conflicts between work and family and bad WLB (Shui et al., 2020). To add to the problem, most career women in China work full time.

Our finding that there was no significant association between gender and job satisfaction is in line with several previous reports involving different samples from several countries. For example, the surveys of 2,584 Canadian physicians (10), of 248 American obstetrician/gynecologists (11), and of other physician samples (12–15), no significant gender differences were found in job satisfaction. In the meantime, some studies have found significant gender differences. For example, Saperstein et al. surveyed 186 Navy family physicians with one self-developed item, and found that males had more positive job satisfaction (16). While in 1,472 doctors from rural areas in West China, the authors reported that female doctors had better job satisfaction than males, based on a self-developed questionnaire (17).

In our study, only 4.21% of the participants were very satisfied with WLB, and 11.45% were satisfied; both were much lower than that reported in other studies. For example, Starmer et al. showed that 17% American pediatricians were very satisfied with their WLB (39); another survey demonstrated that 10.6% US physicians were strongly satisfied and 30.3% satisfied (36); Streu et al. found that 52% U.S. plastic surgeons were satisfied with their WLB (35). The considerable gap suggests much improvement is needed. To improve the status, hospital administrations and policymakers should pay more attention to the job-related risk factors and target risk subgroups, such as surgeons and physicians working in the “miscellaneous departments” (including oncology, reproductive departments, etc.), those with middle or associate senior professional titles, and those with lower-income, and longer working hours.

Furthermore, our finding of no significant gender difference in WLB, from a large sample of Chinese physicians, is also different from most studies in physicians. Literature review shows that four surveys in the U.S. found that female physicians had a significantly lower WLB than male physicians (34–39). Two other U.S. studies found no significant gender difference in physician WLB. In a survey of 127 American faculty surgeons, Baptiste et al. found that there was no significant gender difference in the overall work life balance (mean 2.6 for female vs. 2.9 for male, *p* = 0.3) (37). Similarly, in a sample of 433 physicians in South Dakota, 54.7% of male physicians and 55.4% female physicians were satisfied with their WLB, without significant gender difference (37). Although no gender differences were found in this study, the moderating role of gender in the relationship between organizational behaviors and outcomes should be examined in the future.

Cultural factors, social policies and role expectations have been found to be associated with work-life balance (46, 47). The fact that we did not observe significant gender differences in this sample could also mean more gender equity in family responsibilities in China, especially among married physicians. As suggested by some authors, the rapid economic growth and social progress have pushed more gender equity in employment opportunities and career development (48, 49).

Several limitations about our study need to be mentioned. First, as in all cross-sectional studies, the causal relationship of different factors cannot be established. Second, the participating hospitals were tertiary public hospitals, which often mean they have more cases and also more resources than lower-level hospitals, so the results may not be generalized to all hospitals. Third, although we have some data about the participants' marital status and number of children, we did not collect other important data on their home or personal life, such as the time they spent taking care of chores or other home responsibilities, time for pleasure and relaxation, and these factors are likely associated with WLB (50). Finally, the WLB in our survey was measured by a single item instead of a standardized instrument, which may be limited in reliability and validity.

## Conclusions

In conclusion, while we found that many demographic and job-related factors are significantly associated with job satisfaction and work-life balance, we did not find significant gender differences in either of the assessments between male and female physicians in China, which is different from many other studies. To improve physicians' job satisfaction and WLB in China, interventions should focus on certain specialties and on modifiable factors, such as work hours and income.

## Data Availability Statement

The raw data supporting the conclusions of this article will be made available by the authors, without undue reservation.

## Ethics Statement

The studies involving human participants were reviewed and approved by The Ethics Committee (IEC) of the Emergency General Hospital in Beijing approved the study protocol. The patients/participants provided their written informed consent to participate in this study.

## Author Contributions

DL, YW, FJ, MW, YL, and Y-LT: conceptualization. YW: data curation and investigation. FJ: data analysis. YL: funding acquisition. FJ and Y-LT: methodology. DL: wrote an original draft. DL, YL, and Y-LT: wrote revision & editing. All authors contributed to the article and approved the submitted version.

## Conflict of Interest

The authors declare that the research was conducted in the absence of any commercial or financial relationships that could be construed as a potential conflict of interest.
